# Original Approach to Synthesize TiO_2_/ZnO Hybrid Nanosponges Used as Photoanodes for Photoelectrochemical Applications

**DOI:** 10.3390/ma14216441

**Published:** 2021-10-27

**Authors:** Pedro José Navarro-Gázquez, Maria José Muñoz-Portero, Encarna Blasco-Tamarit, Rita Sánchez-Tovar, Ramon Manuel Fernández-Domene, Jose García-Antón

**Affiliations:** 1 Instituto Universitario de Seguridad Industrial, Radiofísica y Medioambiental (ISIRYM), Universitat Politècnica de València, Camino de Vera s/n, 46022 Valencia, Spain; pednagz@etsii.upv.es (P.J.N.-G.); mjmunoz@iqn.upv.es (M.J.M.-P.); meblasco@iqn.upv.es (E.B.-T.); 2 Departamento de Ingeniería Química, Universitat de Valencia, Av. de las Universitats, s/n, 46100 Burjassot, Spain; rita.sanchez@uv.es (R.S.-T.); ramon.fernandez@uv.es (R.M.F.-D.)

**Keywords:** hybrid nanostructures, nanosponge, titanium dioxide, zinc oxide, photoelectrochemical water splitting

## Abstract

In the present work, TiO_2_/ZnO hybrid nanosponges have been synthesized for the first time. First, TiO_2_ nanosponges were obtained by anodization under hydrodynamic conditions in a glycerol/water/NH_4_F electrolyte. Next, in order to achieve the anatase phase of TiO_2_ and improve its photocatalytic behaviour, the samples were annealed at 450 °C for 1 h. Once the TiO_2_ nanosponges were synthesized, TiO_2_/ZnO hybrid nanosponges were obtained by electrodeposition of ZnO on TiO_2_ nanosponges using different temperatures, times, and concentrations of zinc nitrate (Zn(NO_3_)_2_). TiO_2_/ZnO hybrid nanosponges were used as photoanodes in photoelectrochemical water splitting tests. The results indicate that the photoelectrochemical response improves, in the studied range, by increasing the temperature and the Zn(NO_3_)_2_ concentration during the electrodeposition process, obtaining an increase in the photoelectrochemical response of 141% for the TiO_2_/ZnO hybrid nanosponges electrodeposited at 75 °C with 10 mM Zn(NO_3_)_2_ for 15 min. Furthermore, morphological, chemical, and structural characterization was performed by Field Emission Scanning Electron Microscopy (FE-SEM) with Energy Dispersive X-Ray spectroscopy (EDX), Raman Confocal Laser Spectroscopy, X-Ray Photoelectron Spectroscopy (XPS), and Grazing Incidence X-Ray Diffraction (GIXRD).

## 1. Introduction

The energy crisis is a problem that worries populations around the world. Over time, humans have increasingly become more dependent on energy resources. Currently, the world’s primary energy consumption comes mainly from the use of fossil fuels, whose reserves decrease over time. In addition, their combustion is causing great environmental pollution producing serious consequences such as global warming [1].

The current energy situation leads to unsustainable development, so the need to replace fossil fuels by alternative energy sources that are clean and sustainable. In this context, the idea of hydrogen (H_2_) as a fuel appears, since its combustion reaction only produces water vapor. Furthermore, hydrogen has the highest energy content per unit weight (120 kJ/g) of all fuels [2].

In order to use totally clean and sustainable hydrogen, it must be produced from a renewable energy source. Currently, one of the most promising and novel method is the hydrogen production from the photoelectrochemical water splitting using sunlight. The objective of this method is to use sunlight and a small amount of electrical energy (from another renewable energy source) to get the separation of the water molecule into hydrogen and oxygen. In order to carry out this process, it is necessary to use a semiconductor electrode which may be able to absorb sunlight. In this research, TiO_2_/ZnO hybrid nanosponges have been used as photoanodes for this process.

Titanium oxide (TiO_2_) is one of the most widely studied semiconductor materials for environmental and energy applications owing to its interesting chemical and electronic properties. It is used as a photocatalyst due to its nontoxicity, high stability and photocatalytic activity, cost effectiveness, and its capacity to generate charge by absorbing energy [3,4,5,6,7]. The photocatalytic activity of TiO_2_ depends on the preparation methods and the post-treatment conditions. The synthesis of different TiO_2_ nanostructures is having a great impact because the available active surface increases when boosting the surface-to-volume ratio, thus improving their properties for applications such as catalysis, photocatalysis, and electronic devices [7,8,9]. TiO_2_ nanostructures have been synthesized using different methods such as sol-gel techniques, chemical vapor deposition, vacuum evaporation, hydrothermal methods, anodization, etc. In recent years, self-organized structured oxides formed by anodization of a Ti metallic substrate have attracted considerable interest [10,11,12]. This method possesses the inherent advantage that TiO_2_ nanostructures grow directly on metallic titanium, so it is not necessary to carry out a later stage of compaction or synthesis on a conductive substrate.

The authors obtained a new type of titanium oxide nanostructure (nanosponge) when anodization was performed under hydrodynamic conditions in glycerol/water/ammonium fluoride (NH_4_F) electrolytes. As an advantage, the nanosponge morphology—which consists of a strongly interconnected network of TiO_2_—offers a considerably higher specific area and provides a directly connected path of electrons [11,13,14]. TiO_2_ nanosponges have a significantly rougher morphology than other nanostructures such as TiO_2_ nanotubes, which provides greater electrical conductivity and number of defects. Furthermore, with a nanosponge morphology, there is a displacement of the Fermi level towards the edge of the conduction band, which facilitates the charge separation, resulting in a lower probability of charge recombination [11,13]. In order to transform amorphous TiO_2_ nanosponges prepared by anodization into a crystalline structure, it is necessary to perform an annealing treatment. The anatase phase has a greater mobility of charge carriers than brookite and rutile, so it is the most suitable phase for photoelectrochemical applications [11,14].

One of the most negative aspects of using TiO_2_ as photocatalyst is that it has a wide band gap (3.2 eV for anatase phase, λ = 390 nm), which limits its photocatalytic applications to ultraviolet (UV) irradiation [6]. There are different options to solve this problem such as doping TiO_2_ nanostructures with noble metals [10,15], inserting ions in the TiO_2_ lattice [13,16], using microporous materials [17], and synthesizing hybrid nanostructures [18,19]. Among all these solutions, the most studied option is doping TiO_2_ with noble metals. However, the high cost of noble metals makes it unviable on a large scale. As an alternative, this study proposes the synthesis of TiO_2_/ZnO hybrid nanostructures by electrodeposition of zinc oxide (ZnO) on TiO_2_ nanosponges obtained by anodization.

**Figure 1:**
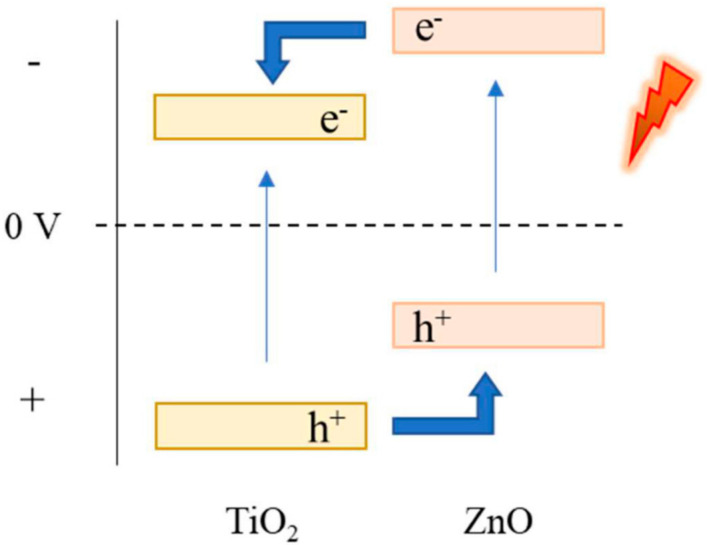
Schematic diagram of the energy level for heterojunction of TiO_2_/ZnO hybrid nanostructures.

ZnO has a conduction band (CB) edge more negative (−0.32 V vs SHE [20]) than TiO_2_ (−0.27 V vs SHE [21]), which allows to improve photoelectrochemical applications of TiO_2_ when TiO_2_/ZnO hybrid nanostructures are formed [22,23,24,25]. Besides, both semiconductors have a similar wide band gap (3.2 eV for anatase-TiO_2_ and 3.4 eV for ZnO), which enables the movement of electrons when they are combined (Figure 1). If TiO_2_/ZnO nanostructures are irradiated, the photoexcited electrons from the CB of ZnO are transferred to the CB of TiO_2_ and, conversely, the holes from the valence band (VB) of TiO_2_ are transferred to the VB of ZnO. This process of transferring electrons and holes can reduce the recombination rate of load carriers, enhancing the photocatalytic activity of photocatalysts [24]. In addition to this, the protective matrix of TiO_2_ reduces the instability problems of ZnO under light irradiation [26,27,28,29].

ZnO incorporation into TiO_2_ nanostructures can be carried out using different techniques such as hydrothermal method [18,25,30], atomic layer deposition [22], and sol-gel method [31,32]. The main problem of these methodologies is that they use severe conditions such as high temperatures, low pressures, or long times. However, TiO_2_/ZnO hybrid nanostructures can also be synthesized by electrodeposition of ZnO on the TiO_2_ nanostructure. The advantage of this method is that it can be easily carried out at low temperatures, at atmospheric pressure, and in short times [3,33].

It has been reported that the introduction of doping atoms into an atomic structure with the corresponding formation of relaxed states is possible, allowing first the formation of disturbed atomic positions subsequently followed by geometric relaxation. In different research, this event was analysed from the Synthetic Growth Concept (SGC). This system allowed modeling films of doping elements formed by precursor species. The simulations involved the optimization of geometries from model systems (templates) and calculations of cohesive energy per atom (E_coh/at_). Cohesive energy is defined as the energy required to divide the system into isolated atomic species. When a system changes its structure or incorporates additional species, there is a change in E_coh/at_ that is equal to the difference between the E_coh/at_ after structural evolution and E_coh/at_ before structural evolution. Structural relaxation occurs as a consequence of bonds breakage and atomic rearrangements. Therefore, the feasibility to intercalate atoms is related to the cohesive energies of the system that contain interstitial defects and the analogous without intercalation defects [34,35,36].

During this study, ZnO was electrodeposited on the TiO_2_ nanosponges using a Zn(NO_3_)_2_ solution as electrolyte. TiO_2_ nanosponges had previously been synthesized by anodization of Titanium (Ti) in a glycerol/water/NH_4_F electrolyte under hydrodynamic conditions. Although ZnO electrodeposition on TiO_2_ nanotubes has been carried out by others authors in previous work [33], it has not been performed on TiO_2_ nanosponges yet. The novelty and originality of this work lies in several factors: (1) This is the first time that TiO_2_/ZnO hybrid nanosponges have been formed using this approach (ZnO electrodeposition on TiO_2_ nanosponges formed by anodization under hydrodynamic conditions). (2) It is based on the importance of performing the ZnO electrodeposition on an ordered structure (crystalline), contrary to what happens in other research where the ZnO electrodeposition is carried out in amorphous structures. (3) The photoelectrochemical activity of TiO_2_/ZnO nanosponges as a function of the ZnO concentration is very different from that obtained when using TiO_2_/ZnO nanotubes synthesized with similar methodologies [26]. The anodization of TiO_2_ nanosponges under hydrodynamic conditions has recently been reported, so there is still room to improve the performance of these nanostructures.

## 2. Experimental Procedure

### 2.1. Preparation of the Photocatalysts

The procedure followed to obtain a nanosponge structure was optimized in previous studies [11,13,14,16]. Titanium rods (8 mm in diameter and 99.3% purity) were polished with 240–4000 silicon carbide (SiC) papers until a mirror surface was reached. After that, samples were immersed in ethanol for 2 min in an ultrasonic bath, rinsed with distilled water, and dried with air. The synthesis of the nanostructures was carried out by electrochemical anodization of titanium rods under hydrodynamic conditions (3000 rpm) in a glycerol/water (60:40 vol %) electrolyte with a concentration of 0.27 M NH_4_F [11]. A two-electrode electrochemical cell with a rotating electrode configuration was used during anodization. The polished Ti sample (0.5 cm^2^) was used as working electrode and a platinum foil as counter electrode. The samples were anodized at room temperature by increasing the potential from 0 to 30 V at a rate of 100 mV·s^−1^, and applying subsequently 30 V for 3 h. During the anodization process, the current density was monitored. Next, samples were rinsed with distilled water and dried with air. Finally, photoelectrodes were annealed at 450 °C for 1 h in air atmosphere with a heating rate of 15 °C/min to transform TiO_2_ nanostructures to the anatase phase. Annealing improves conductivity and lifetime of charge carriers [13,33].

Once the TiO_2_ nanosponges were formed, the ZnO electrodeposition process from a Zn(NO_3_)_2_ solution was carried out, according to the following reactions (Equations (1)–(3)). Equation (4) shows the overall process:

NO_3_^−^ + H_2_O + 2 e^−^ → NO_2_^−^ + 2 OH^−^ (1)

Zn^+2^ + 2 OH^−^ → Zn(OH)_2_ (2)

Zn(OH)_2_ → ZnO + H_2_O (3)

Zn^+2^ + NO_3_^−^ + 2 e^−^ → ZnO + NO_2_^−^ (4)

ZnO formation consists in the ability to generate OH^-^ ions in an aqueous solution containing zinc ions. OH^–^ ions can be generated on the TiO_2_ surface by reducing precursors such as NO_3_^−^, H_2_O, and O_2_. The use of Zn(NO_3_)_2_ makes it possible to have Zn^+2^ ions and nitrates in the solution. The reduction of nitrates to nitrites (Equation (1)) is catalysed by Zn^+2^ ions. Nitrites are adsorbed on the TiO_2_ surface releasing OH^–^ ions, which will precipitate with the Zn^+2^ ions to form the Zn(OH)_2_ complex (Equation (2)). Subsequently, zinc hydroxide spontaneous dehydration occurs to transform it into ZnO (Equation (3)). Equation (4) includes all the steps of the electrodeposition process described above [24,33,37,38].

**Figure 2:**
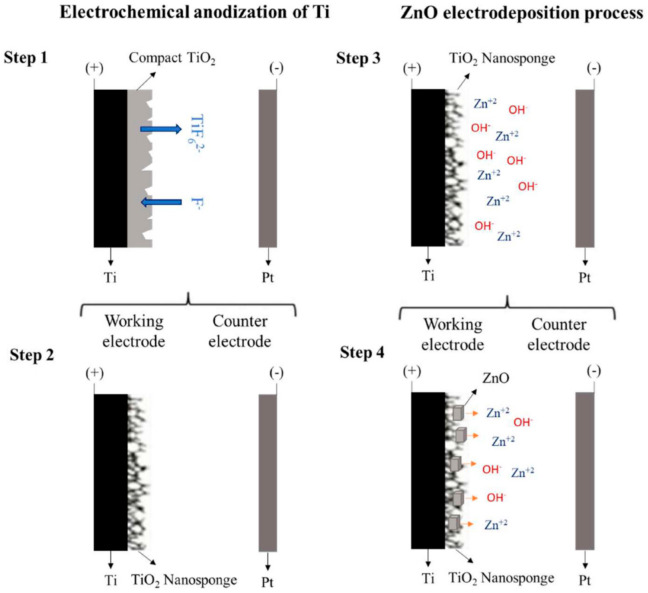
Schematic diagram of growth mechanism of TiO_2_ and ZnO.

ZnO electrodeposition was carried out in a three-electrode electrochemical cell and the temperature was controlled with a thermostatic bath. TiO_2_ nanosponges were used as working electrode, an Ag/AgCl (3 M KCl) electrode was used as reference electrode, and a platinum tip was used as counter electrode. ZnO electrodeposition was carried out at a potential of −0.86 V_Ag/AgCl_ [33,39] using an Autolab PGSTAT302N potentiostat. The growth mechanism of TiO_2_ and ZnO is shown in Figure 2.

The influence of electrodeposition temperature (25 °C, 65 °C, and 75 °C), Zn(NO_3_)_2_ concentration (0.5–10 mM), and electrodeposition time (15, 30, and 60 min) were studied to analyse how these parameters affect the photoelectrochemical activity of the photoelectrodes.

### 2.2. Morphological, Chemical, and Structural Characterization

The morphology of the nanostructures was characterized using Field Emission Scanning Electron Microscopy (FE-SEM) with Energy-Dispersive X-Ray spectroscopy (EDX), which was used to identify the elements that formed the nanostructures applying 20 kV. The equipment used was a Zeiss Ultra-55 Scanning Electron Microscope. In addition, high resolution SEM was also used (Zeiss GeminiSEM 500). Furthermore, the samples were analysed by Raman Confocal Laser Spectroscopy (Witec alpha300 R Confocal Raman Microscope) with a blue laser (488 nm) and a power of 420 μW, to evaluate their crystalline structures.

X-Ray Photoelectron Spectroscopy (XPS) and Grazing Incidence X-Ray Diffraction (GIXRD) were also used to verify the ZnO electrodeposition. On the one hand, all XPS spectra (K-ALPHA Thermo Scientific) were collected using Al-K monochromatized radiation (1486.6 eV) at 3 mA × 12 kV. Scanning step energies of 200 eV were used to measure the whole energy band and 50 eV to selectively measure elements. On the other hand, the apparatus used to obtain GIXRD spectra is a Bruker D8AVANCE diffractometer with Cu radiation operating at 30 mA and 40 kV from 20° to 60° and a grazing incidence of 2°.

### 2.3. Photoelectrochemical Water Splitting Tests

Photoelectrochemical experiments were performed in a cell with a three-electrode configuration using 0.1 M NaOH as electrolyte and a solar simulator (AM 1.5, 100 mW·cm^−2^) connected to a potentiostat (Autolab PGSTAT302N).

The TiO_2_/ZnO hybrid nanosponges, with an area of 0.13 cm^2^ exposed to the test solution were the working electrodes. A platinum tip was used as counter electrode, and an Ag/AgCl (3 M KCl) electrode as reference electrode. For the experiments, a potential sweep was performed from −1 V_Ag/AgCl_ to 0.84 V_Ag/AgCl_ with a scan rate of 2 mV·s^−1^ by chopped light irradiation (60 mV (30 s) in the dark and 20 mV (10 s) with light). Current intensity obtained was normalized with the electrode area.

In addition, in order to compare the photoelectrochemical activity of the nanostructures, current density under illuminated conditions (*i*_max_), increase in current density between dark and light conditions (Δ*i*), and the percentage of improvement in current density under illumination (%*_imp_*) (Equation (5)) were obtained at a potential of 0.6 V_Ag/AgCl_.

(5) $${\%}_{imp}=\frac{i_{TiO2/ZnO}-i_{TiO2}\;}{i_{TiO2}}\cdot 100$$

## 3. Results and Discussion

### 3.1. Synthesis of TiO_2_ Nanosponges

Synthesis of TiO_2_ nanosponges were carried out by electrochemical anodization under hydrodynamic conditions in 0.27 M NH_4_F containing glycerol/water (60:40 vol %) at 30 V for 3 h. Figure S1a (see Supplementary Material) shows the current density versus time obtained during the 3 h of the electrochemical anodization. Three well differentiated stages can be observed. In the first stage, current density decreases with time until a minimum value is reached. The decrease in current density is related to an increase in resistance caused by the formation of a high strength compact TiO_2_ layer on the metallic titanium substrate. Therefore, in this stage, the process is dominated by the electrical resistance of the TiO_2_ compact layer [8,13,14,33].

In the second stage, the current density values increase to a maximum value, and subsequently, they decrease again. This behaviour is caused by the attack of fluorides on the compact surface of TiO_2_, which results in the formation and dissolution of the complex TiF_6_^2−^ (Equation (6)), thus reducing the electrode resistance.

TiO_2_ + 6 F^−^ + 4 H^+^ → [TiF_6_]^−2^ + 2 H_2_O (6)

TiO_2_ dissolution causes the formation of irregular nanopores along the entire electrode surface [8,9,14,27]. The resistance of the ion barrier decreases as the pathways for ionic species in the electrolyte increase. Afterwards, current density decreases again, due to the initial and irregular formation of a TiO_2_ nanostructured layer. This stage represents the transition of a relatively thin compacted TiO_2_ layer at the beginning of a porous and irregular structure formation [8,13,14].

Finally, the third stage is characterized by a progressive increase in the current density until reaching a balance state between the oxide formation at metal/oxide interface and the oxide dissolution at oxide/electrolyte interface. At this stage, the formation and growth of a regular nanosponge layer takes place [8,14,16].

Raman confocal laser spectroscopy was used to evaluate the crystalline structure of the TiO_2_ nanosponges. Figure S1b (see Supplementary Material) shows Raman confocal laser spectra of TiO_2_ nanosponges before and after the heat treatment (450 °C for 1 h). According to other research, only the characteristic peaks of the anatase phase (~145, ~397, ~520 and ~635 cm^−1^) are observed for the annealed nanostructures [16,23,33,40]. No characteristic peaks of any crystalline structure are observed in the nanostructures without annealing. These results indicated that after performing the heat treatment at 450 °C, amorphous TiO_2_ structure transformed into a crystalline structure (anatase phase).

Finally, Figure S1c,d (see Supplementary Material) show the images of the surface of annealed TiO_2_ nanosponges obtained by FE-SEM microscopy at two different magnifications. They confirmed that after electrochemical anodization, uniform, regular, and well-defined TiO_2_ nanostructures were obtained with nanosponge morphology. A very rough surface with a high specific area can be observed in the images.

### 3.2. Synthesis of TiO_2_/ZnO Hybrid Nanosponges Used as Photoanodes

From our knowledge, this is the first time that TiO_2_/ZnO hybrid nanosponges have been synthesized. The first attempt to synthesise TiO_2_/ZnO hybrid nanosponges was developed following the approach employed in previous research, where TiO_2_/ZnO hybrid nanotubes electrodeposited on amorphous TiO_2_ were used [33]. That is, ZnO electrodeposition was performed before the heat treatment. With this synthesis method, the photoelectrochemical activity of the TiO_2_/ZnO hybrid nanosponges electrodeposited with different temperatures (25, 65, and 75 °C), Zn(NO_3_)_2_ concentrations (0.5–10 mM), and times (15, 30, and 60 min) worsened with respect to the TiO_2_ nanosponges, as results shown in Table S1 demonstrate. Table S1 shows i_max_ and Δi for the electrodeposited nanostructures during 15, 30, and 60 min at 25 °C with 1 mM Zn(NO_3_)_2_, and %_imp_ compared to TiO_2_ nanosponges (i_TiO2_ = 0.056 mA·cm^−2^).

There are two possibilities to justify the worse behaviour of TiO_2_/ZnO hybrid nanosponges with respect to TiO_2_ nanosponges: (1) It could be possible that, during the ZnO electrodeposition process under these conditions, the TiO_2_ nanosponges suffered alterations that decreased their photocatalytic activity such as the reduction of Ti^+4^ to Ti^+3^ by the application of a negative potential [16,41] or the random introduction of ZnO in the amorphous TiO_2_ matrix generating stresses and strains when performing the heat treatment [16]. (2) Another possibility is related to the low adherence of ZnO to some substrates [27]; it was unknown if ZnO was introduced inside the TiO_2_ matrix producing stresses and strains, or if the ZnO electrodeposition was not performed correctly due to their low adherence to some substrates. For all these reasons, it was determined that it is not possible to form TiO_2_/ZnO hybrid nanosponges by this approach.

According to these results, it was decided to change the order of the annealing stage to modify the structural orientation of TiO_2_ and not generate stresses during the ZnO intercalation. When electrodepositing before annealing, ZnO is added over an ordered amorphous structure. However, when electrodepositing after annealing, ZnO is added over an ordered crystalline structure. During the next tests, ZnO electrodeposition was carried out after the heat treatment. In this way it was intended to insert ZnO in strategic locations of the TiO_2_ matrix—instead of doing it in messy locations that can generate stresses and strains—improve the ZnO adherence, and increase its stability against negative potentials [16,42]. The influence of time, temperature, and Zn(NO_3_)_2_ concentration during the ZnO electrodeposition process on crystalline TiO_2_ was studied.

#### 3.2.1. Influence of Electrodeposition Time

**Figure 3:**
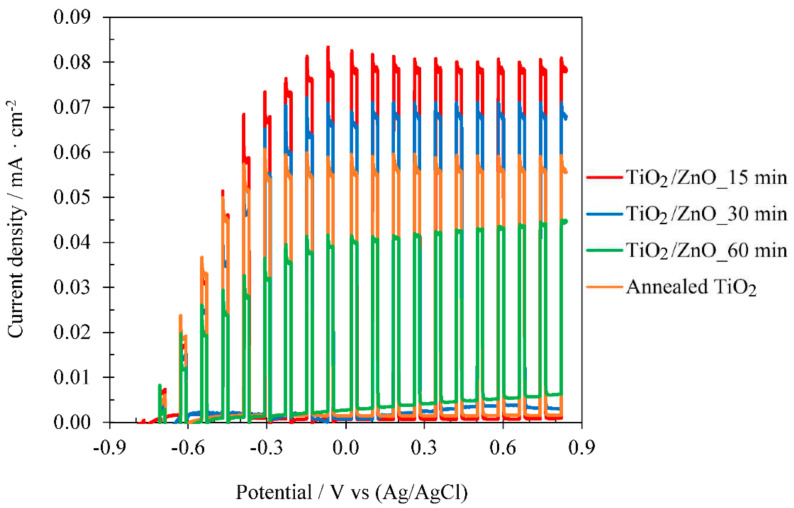
Effect of the electrodeposition time on the photoelectrochemical water splitting tests under dark and illuminated conditions (AM 1.5) for TiO_2_/ZnO hybrid nanosponges electrodeposited at 25 °C with 1 mM Zn(NO_3_)_2_ on crystalline TiO_2_ (comparison with TiO_2_ nanosponges).

In order to analyse the influence of electrodeposition time, ZnO electrodeposition was performed on crystalline TiO_2_ under the optimal conditions obtained previously, i.e: 25 °C and 1 mM Zn(NO_3_)_2_, for 15, 30, and 60 min. Figure 3 shows the current density vs potential obtained during the photoelectrochemical water splitting tests under dark and illuminated conditions (AM 1.5) for TiO_2_ nanosponges and for TiO_2_/ZnO hybrid nanosponges electrodeposited at 25 °C with a Zn(NO_3_)_2_ concentration of 1 mM for 15 min, 30 min and 60 min after the heat treatment.

In Figure 3, it can be observed that by performing a ZnO electrodeposition on crystalline TiO_2_, it is possible to increase the photoelectrochemical performance of TiO_2_/ZnO hybrid nanosponges over that of TiO_2_ nanosponges. The best results were obtained for the nanostructures electrodeposited for 15 min. The photoelectrochemical performance of the nanostructures decreased as the electrodeposition time increased. This factor may be related to the blocking of the TiO_2_ surface caused by a greater amount of electrodeposited ZnO. However, as it will be analysed in a later section (Section 3.2.3 Influence of Zn(NO_3_)_2_ concentration), neither ZnO particles or morphological changes are observed on the nanostructures’ surface. Therefore, by increasing the electrodeposition time, the TiO_2_ nanosponges could undergo changes that decrease their photoelectrocatalytic properties. This factor may be conditioned by a greater reduction from Ti^+4^ to Ti^+3^, when applying a negative potential for a longer time [16,41].

The photocurrent density at 0.6 V_Ag/AgCl_ of the TiO_2_/ZnO hybrid nanosponges electrodeposited for 15 min increased by 37.5% compared to the TiO_2_ nanosponges. Comparing the results obtained in the photoelectrochemical water splitting tests using TiO_2_/ZnO hybrid nanosponges electrodeposited on crystalline TiO_2_ at 25 °C with 1 mM Zn(NO_3_)_2_ for 15 min (i_max_ = 0.077 mA·cm^−2^) with the best results obtained in the previous research [33] using TiO_2_/ZnO hybrid nanotubes electrodeposited on amorphous TiO_2_ at 25 °C with 1 mM Zn(NO_3_)_2_ for 60 min (i_max_ = 0.036 mA·cm^−2^), the photoelectrochemical performance of the TiO_2_/ZnO hybrid nanosponges is approximately 114% higher. Once the time variable was set, studies of the electrodeposition temperature and Zn(NO_3_)_2_ concentration were carried out.

#### 3.2.2. Influence of Electrodeposition Temperature

The influence of electrodeposition temperature was studied next. The electrodeposition temperature plays a very important role in the electrochemical reactions involved in the ZnO electrodeposition. One of the most relevant aspects of ZnO electrodeposition is the influence of pH on the stability of ZnO. The increase in temperature leads to a shift of the solubility and to potential curves towards lower pHs, which favours the ZnO formation. Furthermore, the crystallinity of the electrodeposited ZnO increases with the electrodeposition temperature, which is beneficial for photocatalytic applications [43].

**Figure 4:**
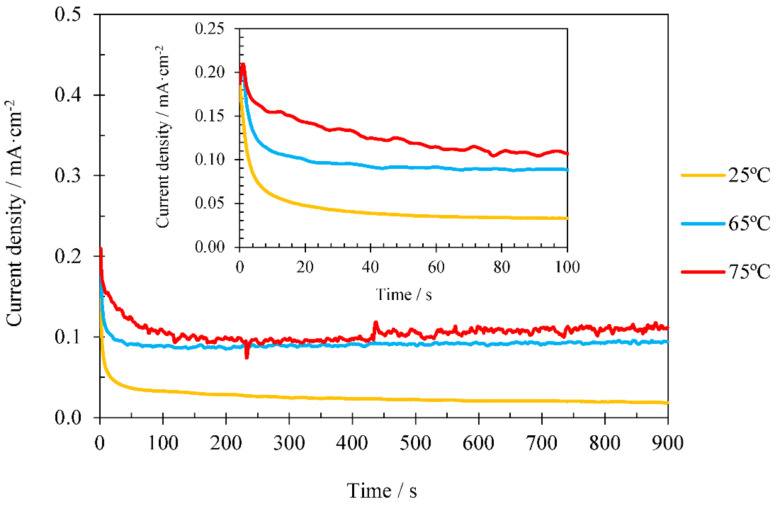
Effect of the electrodeposition temperature for TiO_2_/ZnO hybrid nanosponges during the electrodeposition process with 1 mM Zn(NO_3_)_2_ for 15 min on crystalline TiO_2_, with an insert of the first 100 s.

The formation of ZnO is carried out by the precursor Zn(OH)_2_ at temperatures below 60 °C. Therefore, the ZnO formation is influenced by the dehydration reaction of Zn(OH)_2_ (Equation (3)) at temperatures below 60 °C. However, ZnO is formed directly on the substrate for temperatures above 60 °C, without forming the precursor Zn(OH)_2_. The change in the formation mechanism is attributed to the greater ZnO stability at elevated temperatures [44,45]. The formation reaction of ZnO from Zn(NO_3_)_2_ (Equation (4)) is very slow at low temperatures (25 °C), being extremely fast at temperatures above 70 °C. This is due to the fact that the formation of ZnO (Equation (3)) starts to be more favourable than the formation of Zn(OH)_2_ (Equation (2)) at temperatures above 45 °C, since in Equation (3) the free energy decreases with increasing temperature [37,46,47]. Therefore, in this research it was selected a temperature just below 70 °C (65 °C), another just above (75 °C), and a temperature of 25 °C which can be used as a reference of room temperature. Figure 4 shows, as an example, the current density vs time obtained when performing the ZnO electrodeposition on crystalline TiO_2_ nanosponges for 15 min using 1 mM Zn(NO_3_)_2_. The results obtained follow the same trend in the rest of concentrations.

Figure 4 shows that, in the first seconds of ZnO electrodeposition there are no significant differences in the current density registers for each of the electrodeposition temperatures studied. The initial formation of OH^–^ ions is similar in all cases because the amount of nitrates is the same. For this reason, the nucleation of the ZnO crystals will be similar. However, during the last steps of electrodeposition, current density varies remarkably with temperature. This increase, in addition to being related to a higher conductivity of the electrolyte caused by the increase in temperature, may be caused by a higher amount of ZnO crystals in the TiO_2_ nanostructures and by the size and shape of the crystals [33]. Different studies verified that rising the electrodeposition temperature increases the amount of ZnO present in the nanostructures [33,46,47].

**Figure 5:**
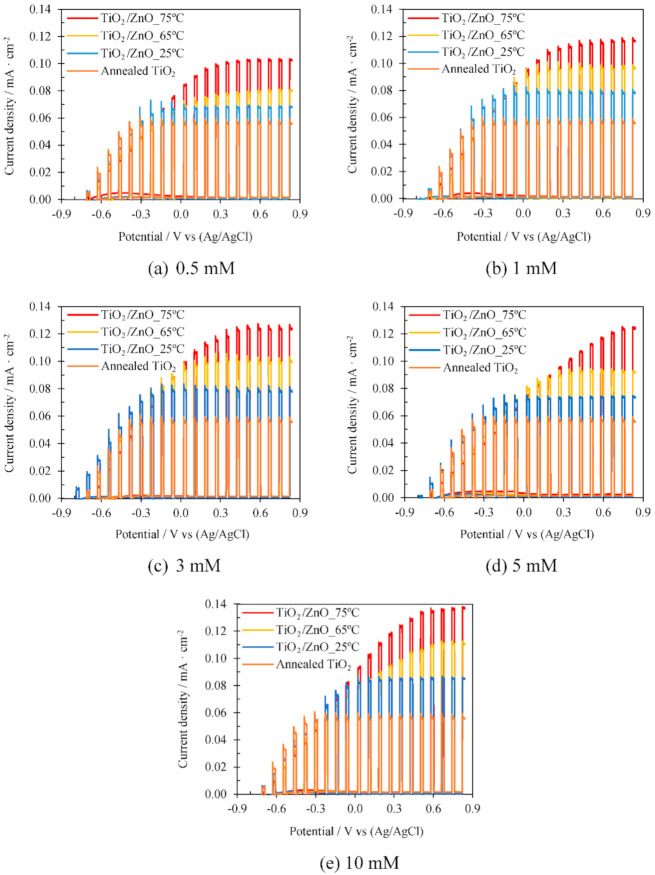
Effect of the electrodeposition temperature on the photoelectrochemical water splitting tests under dark and illuminated conditions (AM 1.5) for TiO_2_/ZnO hybrid nanosponges electrodeposited for 15 min on crystalline TiO_2_ with different Zn(NO_3_)_2_ concentrations: (**a**) 0.5 mM, (**b**) 1 mM, (**c**) 3 mM, (**d**) 5 mM, and (**e**) 10 mM. Comparison with TiO_2_ nanosponges.

Regarding the results obtained during the water splitting tests, Figure 5 shows the photoelectrochemical response of the TiO_2_ nanosponges and the TiO_2_/ZnO hybrid nanosponges electrodeposited on crystalline TiO_2_ for 15 min at 25, 65, and 75 °C for different Zn(NO_3_)_2_ concentrations (0.5, 1, 3, 5, and 10 mM).

Figure 5 shows the results obtained during the photoelectrochemical water splitting tests. The rise and fall of the current density correspond to the lighting switching on and off. Under illumination, a very significant increase in current density is observed over the whole potential range, whereas under dark conditions, current density is practically zero for the whole range of concentrations and temperatures studied. This fact indicates that no undesired oxidation reactions have taken place and that the nanostructures are suitable for the photoelectrochemical water splitting [24,33]. The photoelectrochemical performance of the nanostructures improves when ZnO is incorporated into the crystalline TiO_2_ matrix. Generally, the maximum photocurrent value is reached at 0.6 V_Ag/AgCl_ and it varies almost nothing when applying higher potentials, which is an advantage for photoelectrochemical applications because it is necessary to apply lower polarizations.

In addition, for all the studied concentrations, the higher the electrodeposition temperature, the higher the photocatalytic activity of the TiO_2_/ZnO hybrid nanosponges. The improvement of the photoelectrochemical performance can be influenced by an increase in the amount of ZnO in the nanostructures. ZnO acts as highly efficient charge traps distributed over the entire surface, reducing charge recombination [22,33,41]. The best results of the photoelectrochemical water splitting tests were obtained with TiO_2_/ZnO hybrid nanosponges electrodeposited for 15 min at 75 °C with 10 mM Zn(NO_3_)_2_.

#### 3.2.3. Influence of Zn(NO_3_)_2_ Concentration

**Figure 6:**
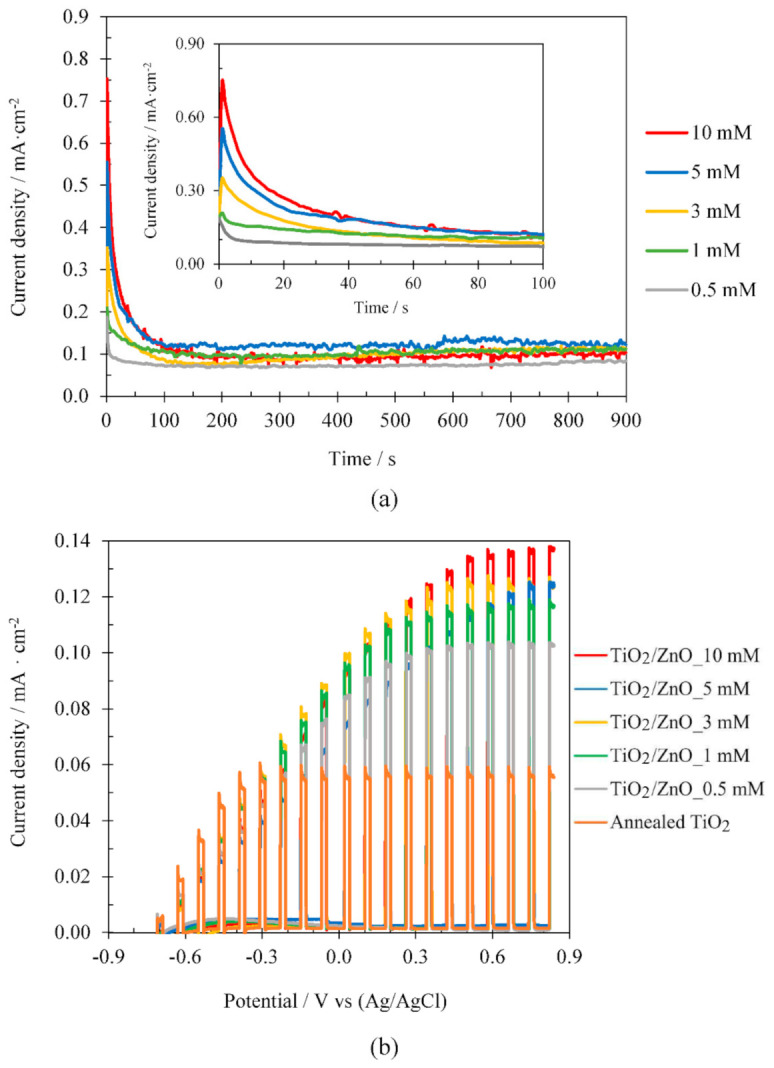
Effect of the Zn(NO_3_)_2_ concentration for TiO_2_/ZnO hybrid nanosponges electrodeposited on crystalline TiO_2_ at 75 °C for 15 min: (**a**) during the electrodeposition process, with an insert of the first 100 s, and (**b**) during the photoelectrochemical water splitting tests under dark and illuminated conditions (AM 1.5) (comparison with TiO_2_ nanosponges).

In this section, the effect of Zn(NO_3_)_2_ concentration on the electrodeposition process was studied. Figure 6 shows, as an example, the results obtained at the temperature that the best photoelectrochemical performances were achieved, i.e., performing the ZnO electrodeposition on crystalline TiO_2_ nanosponges for 15 min at 75 °C with Zn(NO_3_)_2_ concentrations of 0.5, 1, 3, 5 and 10 mM. Figure 6a shows the curve of current density vs time obtained during the ZnO electrodeposition, and Figure 6b shows the photoelectrochemical response of TiO_2_ nanosponges and TiO_2_/ZnO hybrid nanosponges electrodeposited under the conditions mentioned above.

On the one hand, Figure 6a shows that in the first seconds of ZnO electrodeposition, the maximum current density value increases with the Zn(NO_3_)_2_ concentration [33,37], while in the final electrodeposition instants no significant differences are found. The current density decreases from a maximum value, which is related to the reduction of nitrates to nitrites and the OH^–^ ions formation on the TiO_2_ surface. Nitrates reductions enable the ZnO formation from Zn^+2^ and OH^–^ ions, according to the reactions described in Equations (1)–(4). If Zn(NO_3_)_2_ concentration increases, more nitrates will be reduced in the proximity of TiO_2_ and, therefore, more ZnO will be formed [33].

On the other hand, in Figure 6b it can observed that, by increasing the Zn(NO_3_)_2_ concentration from 0.5 mM to 10 mM, the photoelectrochemical performance of the nanostructures considerably improves. The best results are obtained when using a Zn(NO_3_)_2_ concentration of 10 mM because the higher nucleation of ZnO crystals increases the photocatalytic activity of the nanostructures and the useful life of the excited electrons, reducing recombination processes [33,48,49,50].

Table 1 shows the results obtained during the water splitting tests at a potential of 0.6 V_Ag/AgCl_ under illumination, for the TiO_2_/ZnO hybrid nanosponges electrodeposited on crystalline TiO_2_ for 15 min at 25, 65, and 75 °C with Zn(NO_3_)_2_ concentrations of 0.5, 1, 3, 5 and 10 mM. The improvement percentage (%_imp_) has been calculated according to Equation (5).

In Table 1 it can be observed that, for all the studied temperatures, the general trend is an increase in current density with the Zn(NO_3_)_2_ concentration. In addition, the difference of the current density between illuminated and dark conditions was similar to the net current density in illuminated conditions. Therefore, the photoelectrodes were stable in the studied potential range. The best results were obtained at 10 mM Zn(NO_3_)_2_.

The ZnO electrodeposition on crystalline TiO_2_ nanosponges improves substantially the photoelectrochemical performance of the nanostructures. Under the best studied conditions—performing the ZnO electrodeposition on crystalline TiO_2_ nanosponges for 15 min at 75 °C with 10 mM Zn(NO_3_)_2_—it was possible to improve the photoelectrochemical response during the water splitting tests by 141% with respect to TiO_2_ nanosponges. Compared to the best results obtained in the previous research [33]—using TiO_2_/ZnO nanotubes electrodeposited for 60 min at 25 °C with 1 mM Zn(NO_3_)_2_—the improvement in the photoelectrochemical performance is 275%.

**Figure 7:**
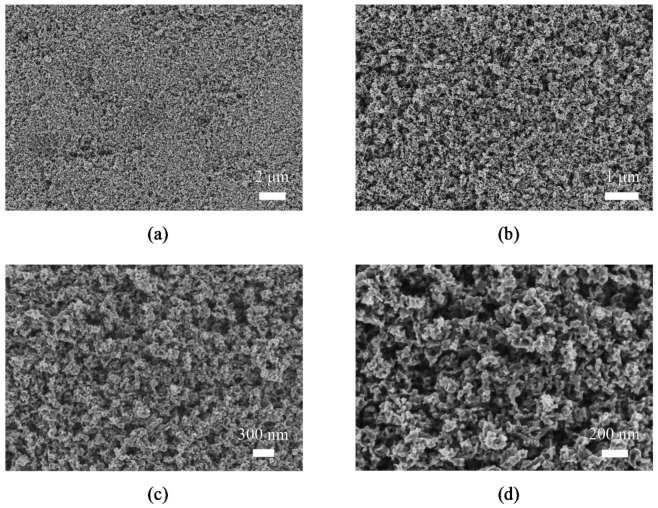
High resolution FE-SEM images of the TiO_2_/ZnO hybrid nanosponges electrodeposited on crystalline TiO_2_ for 15 min at 75 °C with 10 mM Zn(NO_3_)_2_ at (**a**) 5000×, (**b**) 15,000×, (**c**) 30,000×, and (**d**) 50,000×.

Figure 7 shows, as an example, the general appearance of the surface of TiO_2_/ZnO hybrid nanosponges electrodeposited at 75 °C with 10 mM Zn(NO_3_)_2_ for 15 min. FE-SEM images at four different magnifications are shown.

In Figure 7 it can be observed that the general appearance of the nanostructures is the same as the one observed for the TiO_2_ photocatalysts. A nanosponge-shaped nanostructure is seen without the presence of anomalous particles on its surface. In the range of concentrations and temperatures studied, ZnO particles were not appreciated which could hide the active surface of the TiO_2_ nanosponge. This indicates that no ZnO agglomerations occurred during the electrodeposition process. ZnO presence in the nanostructures will be justified in the Section 3.4.

### 3.3. Statistical Analysis

In order to analyse the influence of Zn(NO_3_)_2_ concentration and electrodeposition temperature in more detail, a statistical study of these variables and their interaction was carried out. Table S2 shows the results from the statistical analysis of the current density obtained during the photoelectrochemical water splitting tests under illumination at a potential of −0.6 V_Ag/AgCl_, using TiO_2_/ZnO hybrid nanosponges electrodeposited on crystalline TiO_2_ for 15 min at different temperatures (25, 65, and 75 °C) and Zn(NO_3_)_2_ concentrations (0.5, 1, 3, 5, and 10 mM).

The *p*-values lower than 0.05 correspond to the effect of the Zn(NO_3_)_2_ concentration (0.0057) and the temperature (0.0000), so that both parameters are statistically significant. This means that during the ZnO electrodeposition process, as the temperature and the Zn(NO_3_)_2_ concentration increase (within the studied range), the photocurrent density of the TiO_2_/ZnO hybrid nanosponges for water splitting photoelectrochemical process also increases. The rest of the factors had *p*-values considerably greater than 0.05, so it was determined that they were not statistically significant.

Therefore, in order to synthesize the TiO_2_/ZnO nanosponges offering the highest photoelectrochemical performance, it is necessary to increase the temperature and the Zn(NO_3_)_2_ concentration during the electrodeposition process up to the maximum studied values (75 °C and 10 mM, respectively). In the next section, it will be verified whether the increase in photoelectrochemical performance as the temperature and the Zn(NO_3_)_2_ concentration increase is related to an increase in the ZnO amount present in the nanostructures.

### 3.4. Chemical Composition

In order to verify the ZnO presence in the TiO_2_ nanosponges, X-Ray Photoelectron Spectroscopy (XPS), Energy-Dispersive X-Ray spectroscopy (EDX), and Grazing Incidence X-Ray Diffraction (GIXRD) were used.

First, the presence of ZnO was verified using the XPS technique. XPS was used to determinate the bonding states. Figure S2 shows the XPS spectra of the TiO_2_ nanosponges and TiO_2_/ZnO hybrid nanosponges electrodeposited for 15 min at 75 °C with a Zn(NO_3_)_2_ concentration range between 1 and 10 mM. It can be observed that, in all XPS spectra, the peak corresponding to C1s at position 284.6 eV appears, which is a fact related to the organic species involved in the preparation (glycerol) and to the contaminants adsorbed in the nanostructures. C1s peak position can be used as a reference for the rest of the peaks involved in the XPS spectra [23,51]. As expected, peaks related to Zn are only observed in TiO_2_/ZnO hybrid nanostructures.

**Figure 8:**
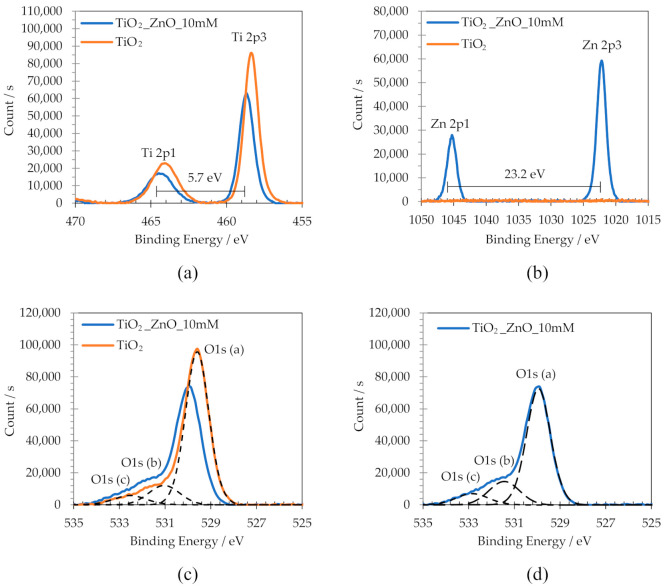
High-resolution XPS spectra of (**a**) Ti2p, (**b**) Zn2p, (**c**) and (**d**) O1s, of TiO_2_ nanosponges and TiO_2_/ZnO hybrid nanosponges electrodeposited for 15 min at 75 °C with 10 mM Zn(NO_3_)_2_ after the heat treatment.

Regions offering the greatest interest are discussed in more detail below. Figure 8 shows, as an example, high-resolution spectra of Ti2p, Zn2p, and O1s, of TiO_2_ nanosponges and TiO_2_/ZnO hybrid nanosponges electrodeposited on crystalline TiO_2_ for 15 min at 75 °C with 10 mM Zn(NO_3_)_2_.

First, Figure 8a shows the two characteristic Ti2p peaks that form photoelectrons 2p_1/2_ and 2p_3/2_ at positions 464.1 eV and 458.4 eV, respectively. Both the position and the separation between them (5.7 eV) are associated to the valence state of Ti^+4^ and, therefore, they confirm the presence of TiO_2_ in the nanostructures. The chemical shift of the peaks is due to the charge imbalance caused by ZnO introduction into the TiO_2_ nanostructure since Zn (1.6 eV·e^−1^) has higher Pauling electronegativity values than Ti (1.5 eV·e^−1^) [23,49,52]. In general, the binding energy values (eV) increase as the electronegativity of the bonded elements increases. There are reference values in literature for the binding energies of many compounds, which can vary between 0.1 and 10 eV depending on the chemical element to which it is bound [53]. In addition, it is also observed that for TiO_2_/ZnO hybrid nanostructures the intensity of the peaks decreases due to a lower TiO_2_ proportion present in the nanostructures.

Secondly, Figure 8b shows the Zn2p characteristic region. The Zn2p high-resolution spectrum shows that in the TiO_2_/ZnO hybrid nanosponges the doublet peaks corresponding to Zn2p_1/2_ and Zn2p_3/2_ can be appreciated at approximately 1045.2 and 1022 eV, respectively, and the distance between them is about 23.2 eV. These results proved that Zn species are in its Zn^+2^ oxidation state, confirming the presence of ZnO in the TiO_2_/ZnO nanostructures [23,25,49,51,52,54].

Finally, Figure 8c,d shows the high-resolution spectra for O1s. On the one hand, Figure 8c shows the deconvolutions carried out for TiO_2_ nanosponges, called O1s_a, O1s_b and O1s_c. The main component (O1s_a) has a binding energy of 529.6 eV and it is assigned with the lattice oxygen (L_o_) of TiO_2_, that is, corresponds to the O^2-^ atoms with total coordination with Ti^+4^ in the TiO_2_ lattice. The second component (O1s_b) has a binding energy of 531.1 eV and it can be associated with oxygen vacancies (V_o_) in the TiO_2_ network. The third peak (O1s_c) has a binding energy of 532.5 eV and it is related to the oxygen atoms of water, organic species, or O_2_ that react on the surface [55,56,57,58,59]. Organic species could come from chemical compounds used during the synthesis or from contaminants adsorbed on the surface. Furthermore, it is observed again that the O1s peaks of TiO_2_/ZnO hybrid nanosponges may be displaced by the ZnO introduction into the nanostructure. On the other hand, Figure 8d shows the deconvolutions carried out for TiO_2_/ZnO hybrid nanosponges electrodeposited for 15 min at 75 °C with 10 mM Zn(NO_3_)_2_. The TiO_2_/ZnO hybrid nanosponges show the same O1s deconvolution peaks as the TiO_2_ nanosponges.

Table 2 shows the atomic ratio (%_At_) obtained from the XPS analysis for TiO_2_ nanosponges and TiO_2_/ZnO hybrid nanosponges electrodeposited at 75 °C for 15 min with Zn(NO_3_)_2_ concentration of 1, 3, 5, and 10 mM. It shows the atomic ratios of O1s (O1s_a, O1s_b, and O1s_c), Ti^+4^, and Zn^+2^. The remaining elements present in the nanostructures have been omitted because they are contaminants adsorbed on the surface that can affect the analysis of the results.

Table 2 shows that the atomic ratio of Zn^+2^ increases with the Zn(NO_3_)_2_ concentration. So, the higher the Zn(NO_3_)_2_ concentration is, the greater the amount of electrodeposited ZnO becomes in the studied concentration range. Furthermore, an increase in the concentration of oxygen vacancies (O1s_b) is observed as the ZnO ratio increases. By inserting ZnO into the TiO_2_ network, a greater number of defects are generated, which is beneficial for photoelectrochemical applications. Oxygen vacancies act as charge donors, decreasing the recombination processes, and improving the charge transfer [60]. These results are supported by those obtained during the photoelectrochemical water splitting tests, where the photocatalytic activity of the nanostructures increased with the Zn(NO_3_)_2_ concentration, that is, increasing the amount of electrodeposited ZnO.

**Figure 9:**
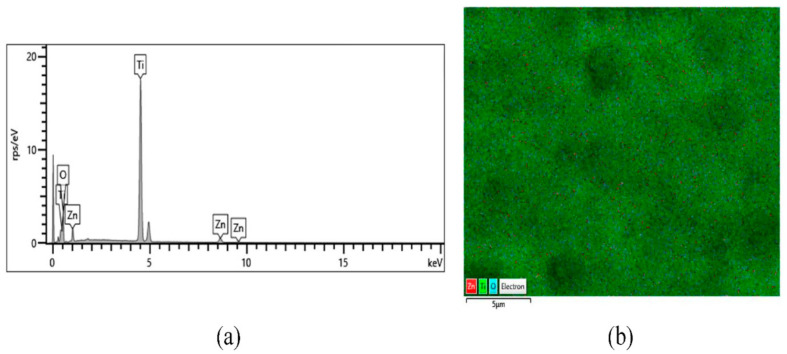
EDX analysis of the TiO_2_/ZnO hybrid nanosponges electrodeposited for 15 min at 75 °C with 10 mM Zn(NO_3_)_2_ concentration on crystalline TiO_2_: (**a**) EDX spectrum of elements and (**b**) EDX mapping of the surface differentiating elements by colours.

Once the presence of ZnO has been verified, an EDX analysis was carried out to detect its distribution and quantity depending on the electrodeposition temperature and Zn(NO_3_)_2_ concentration. Figure S3 shows EDX mapping of the TiO_2_/ZnO hybrid nanosponges electrodeposited at 75 °C for 15 min with Zn(NO_3_)_2_ concentrations of 1, 3, 5, and 10 mM. Figure 9 shows, as an example, the results of the nanostructures—obtained by EDX—that have presented the highest photoelectrochemical response, i.e., the TiO_2_/ZnO hybrid nanosponges electrodeposited on crystalline TiO_2_ at 75 °C with 10 mM Zn(NO_3_)_2_ for 15 min. It has been determined that EDX analysis detected the presence of Zn in the nanostructures, and that it is well distributed over the whole surface without forming large agglomerations. Carbon elements have been omitted from the analysis because they are residual.

In order to study how the temperature and the Zn(NO_3_)_2_ concentration affected the ZnO amount deposited on crystalline TiO_2_ nanostructures, Table 3 shows the compositions obtained by EDX of TiO_2_ nanosponges and TiO_2_/ZnO hybrid nanosponges electrodeposited for 15 min at different temperatures (25 °C, 65 °C and 75 °C) and Zn(NO_3_)_2_ concentrations (1, 5 and 10 mM). The improvement percentage obtained from Equation (5) has also been added.

Table 3 shows, on the one hand, an increase of the Zn amount in the nanostructures with the temperature and the Zn(NO_3_)_2_ concentration. Furthermore, in O/Ti column it is observed that the TiO_2_ nanosponges have an O/Ti atomic ratio of 1.6, while in the TiO_2_/ZnO hybrid nanosponges the ratio is between 1.8 and 2, approximately. The increase in the O/Ti atomic ratio can be explained by the presence of O atoms that are bound to Zn. If Zn had not been added in its oxidation state (ZnO), the O/Ti ratio would have remained constant in all the formed nanostructures [33,48,61,62]. On the other hand, in the percentage improvement column (%_imp_) it is confirmed that by increasing the ZnO amount in the TiO_2_/ZnO hybrid nanosponges—in the range studied—the photoelectrochemical activity of the nanostructures increases.

The atomic ratios obtained using the EDX technique are not comparable to those obtained from XPS because the results are influenced by the energies used and the degree of penetration of the incident elements. However, the same conclusions have been reached taking into account the data extracted from both techniques.

**Figure 10:**
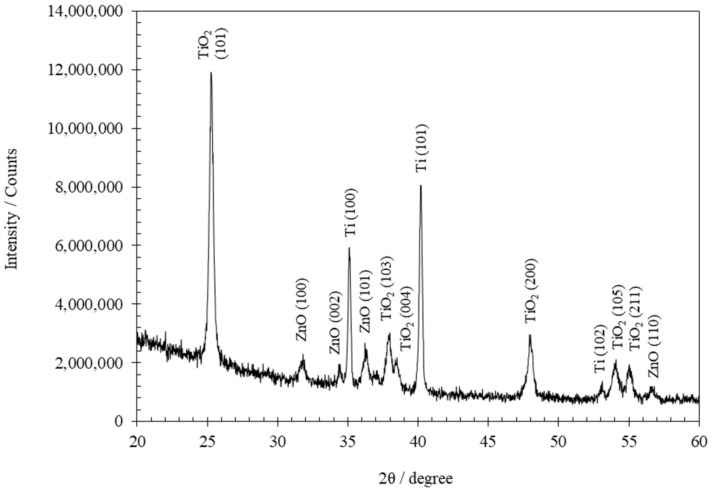
GIXRD pattern of the TiO_2_/ZnO hybrid nanosponges electrodeposited for 15 min at 75 °C with 10 mM Zn(NO_3_)_2_ after the heat treatment.

Finally, an X-Ray Diffraction study is carried out. However, since there are two layers overlaps—a layer of ZnO over another of TiO_2_—it is necessary to perform the Grazing Incidence X-Ray Diffraction (GIXRD) technique. Figure 10 shows, as an example, grazing incidence GIXRD pattern of TiO_2_/ZnO hybrid nanosponges electrodeposited on crystalline TiO_2_ at 75 °C with 10 mM Zn(NO_3_)_2_ for 15 min.

The peaks obtained in the GIXRD analysis correspond to TiO_2_ and ZnO. Figure 10 shows a very intense peak located at 25.3° corresponding to the main characteristic reflection of the anatase TiO_2_ phase (101) (according to JCPDS 21-1272) [63,64]. Furthermore, four of the main crystallographic peaks of the wurtzite structure of ZnO (according to JCPDS 36-1451) appear at 31.8°, 34.4°, 36.2°, and 56.7°, corresponding to crystallographic planes (100), (002), (101), and (110), respectively [17,64,65,66].

After completing the morphological, chemical, and structural analysis of the TiO_2_/ZnO hybrid nanostructures, it was verified that the ZnO addition was carried out successfully by performing the electrodeposition process on TiO_2_ nanosponges with crystalline structure for 15 min in the range of concentrations and temperatures studied.

## 4. Conclusions

In this research, TiO_2_/ZnO hybrid nanosponges have been synthesized for the first time. The nanostructures were synthesized by anodization of titanium under hydrodynamic conditions and, subsequently, ZnO electrodeposition was carried out by modifying the Zn(NO_3_)_2_ concentration, time, and temperature.

The photocatalytic activity of the hybrid nanostructures increased considerably with respect to TiO_2_ nanosponges. The photoelectrochemical response improved by decreasing the electrodeposition time from 60 to 15 min, and by increasing the Zn(NO_3_)_2_ concentration and the electrodeposition temperature throughout the studied range. The best results in the photoelectrochemical water splitting tests were obtained when performing the ZnO electrodeposition for 15 min at 75 °C with a Zn(NO_3_)_2_ concentration of 10 mM. Under these conditions, it was possible to improve the photoelectrochemical response by 141% with respect to the crystalline TiO_2_ nanosponges, and by 275% with respect to the TiO_2_/ZnO hybrid nanotubes that showed the best results in the photoelectrochemical water splitting tests from previous research.

The statistical analysis of the electrodeposited nanostructures after annealing showed that both electrodeposition temperature and the Zn(NO_3_)_2_ concentration were statistically significant parameters.

FE-SEM images showed a sponge-shaped nanostructure without the presence of anomalous particles, so ZnO was introduced into the TiO_2_ matrix without forming agglomerations. Furthermore, it was observed by EDX that the ZnO amount present in the nanostructures increases as the Zn(NO_3_)_2_ concentration and electrodeposition temperature increased, and that the ZnO was well distributed over the entire surface of the TiO_2_/ZnO hybrid nanosponges.

XPS analysis determined the presence of the doublet peaks corresponding to Zn2p_1/2_ and Zn2p_3/2_ at approximately 1045.2 and 1022 eV, proving that Zn species were in their Zn^+2^ oxidation state, which confirms the presence of ZnO in the TiO_2_/ZnO hybrid nanosponges.

Finally, the presence of ZnO was also verified using the GIXRD technique. The crystallographic peaks of the wurtzite structure of ZnO at 31.8° (100), 34.4° (002), 36.2° (101) and 56.7° (110) could be observed.

## Figures and Tables

**Table 1:** Current density at 0.6 V_Ag/AgCl_ under illuminated conditions (i_max_), increase in current density at 0.6 V_Ag/AgCl_ between dark and light conditions (Δi), and percentage improvement in current density at 0.6 V_Ag/AgCl_ under illumination (%_imp_) with respect to the TiO_2_ nanosponges, for TiO_2_/ZnO hybrid nanosponges electrodeposited on crystalline TiO_2_ for 15 min.

Temperature (ºC)	Concentration (mM)	i max (mA·cm^-2^)	Δi (mA·cm^-2^)	%_imp_
25	0.5	0.068	0.067	21.43
	1	0.077	0.077	37.50
	3	0.078	0.077	39.29
	5	0.073	0.072	30.36
	10	0.085	0.085	51.79
65	0.5	0.093	0.093	66.07
	1	0.096	0.096	71.43
	3	0.099	0.098	76.79
	5	0.103	0.102	83.93
	10	0.109	0.105	94.64
75	0.5	0.103	0.102	83.93
	1	0.114	0.113	103.57
	3	0.123	0.122	119.64
	5	0.117	0.112	108.93
	10	0.135	0.134	141.07

**Table 2:** Atomic ratio of O1s (O1s_a, O1s_b, and O1s_c), Ti^+4^, and Zn^+2^ for TiO_2_ nanosponges and TiO_2_/ZnO hybrid nanosponges electrodeposited for 15 min at 75 °C with Zn(NO_3_)_2_ concentration of 1, 3, 5, and 10 mM.

	TiO_2_ (%_At_)	TiO_2__1 mM (%_At_)	TiO_2__3 mM (%_At_)	TiO_2__5 mM (%_At_)	TiO_2__10 mM (%_At_)
O1s_a	58.46	49.64	47.34	46.70	41.50
O1s_b	9.68	10.55	12.11	13.05	19.01
O1s_c	4.94	6.35	6.58	5.86	7.42
Ti^+4^	26.91	25.25	24.72	22.07	18.32
Zn^+2^	0.00	8.20	9.25	12.32	13.77

**Table 3:** EDX results of TiO_2_ nanosponges and TiO_2_/ZnO hybrid nanosponges electrodeposited at different temperatures and Zn(NO_3_)_2_ concentrations, and %_imp_.

Sample	Ti		O		Zn		O/Ti	%_Imp_
	Weight Ratio	Atomic Ratio	Weight Ratio	Atomic Ratio	Weight Ratio	Atomic Ratio	Atomic Ratio	
TiO_2_	65.49	38.79	34.51	61.21	0.00	0.00	1.58	-
TiO_2_/ZnO_25ºC_10mM	62.23	35.85	36.98	63.81	0.79	0.34	1.78	51.79
TiO_2_/ZnO_65ºC_10mM	59.03	34.04	37.30	64.40	3.67	1.55	1.89	94.64
TiO_2_/ZnO_75ºC_10mM	57.81	34.43	35.04	62.45	7.15	3.12	1.81	141.07
TiO_2_/ZnO_75ºC_5mM	58.08	34.10	36.07	63.39	5.85	2.51	1.86	108.93
TiO_2_/ZnO_75ºC_1mM	57.56	32.92	38.11	65.26	4.33	1.82	1.98	103.57

## Data Availability

Not applicable.

## Supplementary Materials

The following are available online at https://www.mdpi.com/article/10.3390/ma14216441/s1, Figure S1: (a) Current density versus time during anodization of Ti in 0.27 M NH4F containing glycerol/water (60:40 vol. %) at 30 V for 3 h. (b) Raman spectra of TiO2 nanosponges before and after the heat treatment at 450 ºC for 1 h. FE-SEM images of the annealed TiO2 nanosponges at (c) 5000 X, and (d) 15000 X., Figure S2: XPS spectra of TiO2 nanosponges and TiO2/ZnO hybrid nanosponges electrodeposited on crystalline TiO2 for 15 minutes at 75 ºC with different Zn(NO3)2 concentrations., Figure S3: EDX mapping of TiO2/ZnO hybrid nanosponges electrodeposited for 15 min at 75 ºC with Zn(NO3)2 concentration of (a) 1, (b) 3, (c) 5, and (d) 10 mM., Table S1: Current density at 0.6 VAg/AgCl under illuminated conditions (imax), increase in current density at 0.6 VAg/AgCl between dark and light conditions (Δi), and percentage improvement in current density at 0.6 VAg/AgCl under illumination (%improvement) with respect to the TiO2 nanosponges, for TiO2/ZnO hybrid nanosponges electrodeposited on amorphous TiO2 at 25 ºC with 1 mM Zn(NO3)2, Table S2: Analysis of variance for density current (mA·cm-2) of the individual factors of Zn(NO3)2 concentrations and temperature, the interaction between them, and their quadratic effects.
