# Supplementary material for: Original Approach to Synthesize TiO2/ZnO Hybrid Nanosponges Used as Photoanodes for Photoelectrochemical Applications
Source: Materials (Basel). 2021 Oct 27;14(21):6441. doi: 10.3390/ma14216441 (PMC8585194; doi:10.3390/ma14216441)
Supplement: Supplementary file 1 [file materials-14-06441-s001.zip › materials-1426070-supplementary.pdf]

materials

MDPI

Supplementary Materials: Original Approach to Synthesize TiO_2/ZnO Hybrid Nanosponges Used as Photoanodes for Photoelectrochemical Applications

P. J. Navarro-Gázquez [1], M. J. Muñoz-Portero [1], E. Blasco-Tamarit [1], R. Sánchez-Tovar [2], R. M. Fernández-Domene [2] and J. García-Antón [1,*]

[1] Instituto Universitario de Seguridad Industrial, Radiofísica y Medioambiental (ISIRYM). Universitat Politècnica de València. Camino de Vera s/n, 46022 Valencia, Spain; pednagz@etsii.upv.es (P.J.N.-G.); mjmunoz@iqn.upv.es (M.J.M.-P.); meblasco@iqn.upv.es (E.B.-T.); jgarciaa@iqn.upv.es (J.G.-A.)

[2] Departamento de Ingeniería Química, Universitat de Valencia, Av. de las Universitats, s/n, 46100 Burjassot, Spain; rita.sanchez@uv.es (R.S.-T.); ramon.fernandez@uv.es (R.M.F.-D.)

* Correspondence: jgarciaa@iqn.upv.es ; Tel.: +34-96-387-76-32; Fax: 34-96-387-76-39

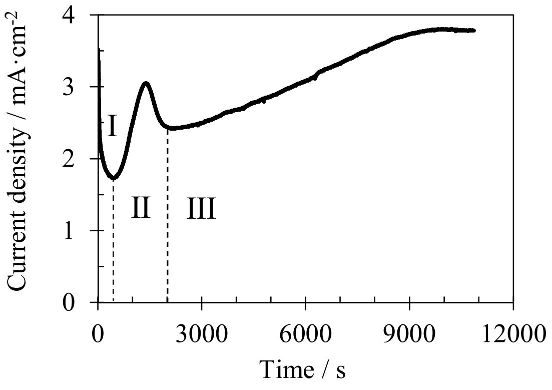

(a)

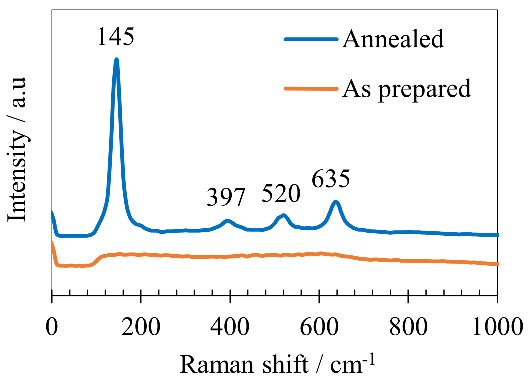

(b)

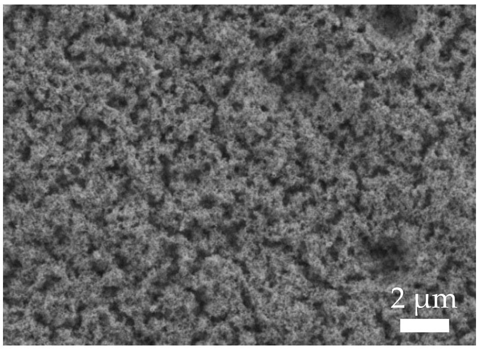

(c)

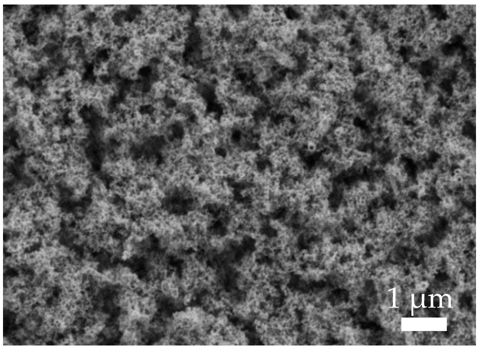

(d)

Figure S1. (a) Current density versus time during anodization of Ti in 0.27 M NH_4F containing glycerol/water (60:40 vol. %) at 30 V for 3 h. (b) Raman spectra of TiO_2 nanosponges before and after the heat treatment at 450 ºC for 1 h. FE-SEM images of the annealed TiO_2 nanosponges at (c) 5000 X, and (d) 15000 X.

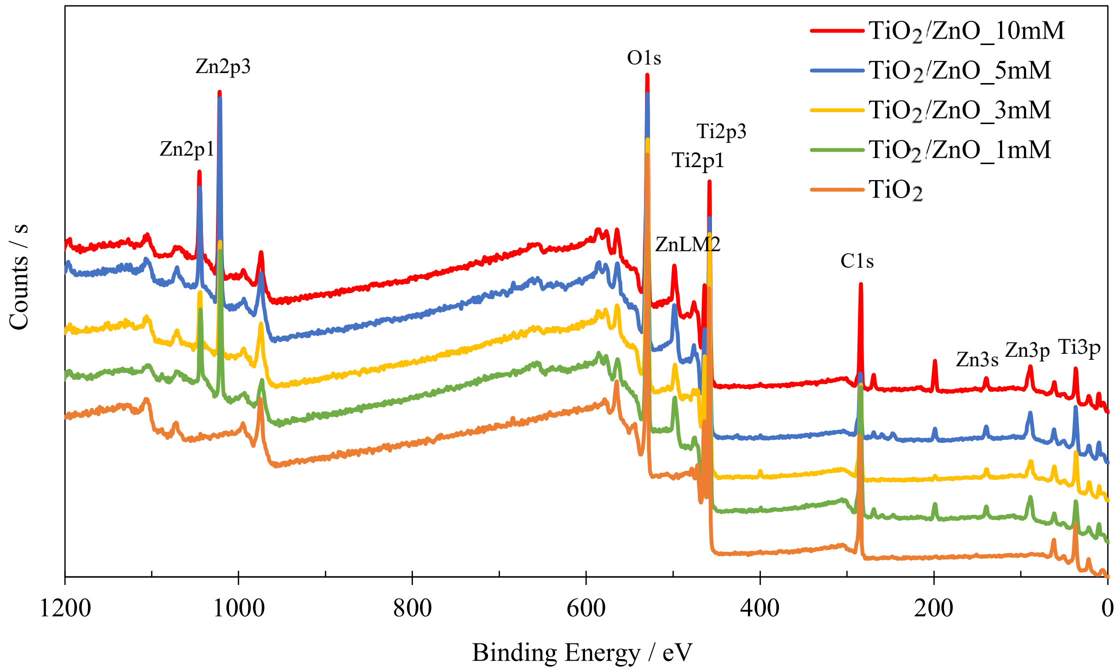

Figure S2. XPS spectra of TiO_2 nanosponges and TiO_2/ZnO hybrid nanosponges electrodeposited on crystalline TiO_2 for 15 minutes at 75 ºC with different $Zn(NO_3)_2$ concentrations.

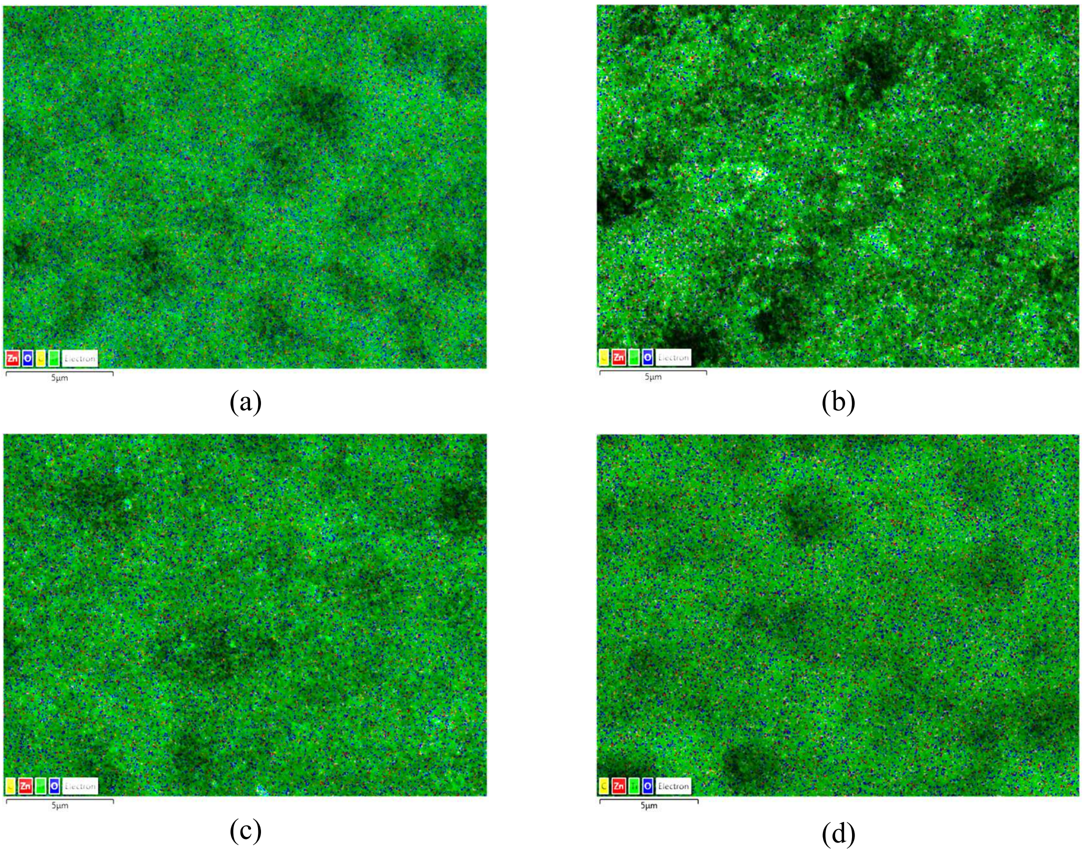

Figure S3. EDX mapping of TiO_2/ZnO hybrid nanosponges electrodeposited for 15 min at 75 ºC with $Zn(NO_3)_2$ concentration of (a) 1, (b) 3, (c) 5, and (d) 10 mM.

Table S1. Current density at 0.6 $V_{Ag/AgCl}$ under illuminated conditions (i_{max}), increase in current density at 0.6 $V_{Ag/AgCl}$ between dark and light conditions (Δi), and percentage improvement in current density at 0.6 $V_{Ag/AgCl}$ under illumination ($\%_{improvement}$) with respect to the TiO_2 nanosponges, for TiO_2/ZnO hybrid nanosponges electrodeposited on amorphous TiO_2 at 25 ºC with 1 mM $Zn(NO_3)_2$.

Time (min)	i_{max} (mA·cm^{-2})	Δi (mA·cm^{-2})	$\%_{improvement}$
15	0.029	0.028	-48.21
30	0.024	0.023	-57.14
60	0.014	0.012	-75.00

Table S2. Analysis of variance for density current (mA·cm^{-2}) of the individual factors of $Zn(NO_3)_2$ concentrations and temperature, the interaction between them, and their quadratic effects.

	Sum of squares	Gl	Mean square	F_{ratio}	p-Value
$Zn(NO_3)_2$	0.00016737	1	0.00016737	9.28	0.0057
Temperature	0.00197606	1	0.00197606	109.55	0.0000
$Zn(NO_3)_2{}^2$	5.88984E-7	1	5.88984E-7	0.03	0.8582
$Zn(NO_3)_2$·T	2.58574E-7	1	2.58574E-7	0.01	0.9057
Temperature^2	0.0000449854	1	0.0000449854	2.49	0.1279
Residual error	0.000414857	23	0.0000180372		
Total	0.00264598	29			

Statistically significant factor when p-Value is lower to 0.05.
